# Protic Ionic Liquids as Efficient Solvents in Microwave-Assisted Extraction of Rhein and Emodin from *Rheum palmatum* L.

**DOI:** 10.3390/molecules24152770

**Published:** 2019-07-30

**Authors:** Yunchang Fan, Zeyu Niu, Chen Xu, Lei Yang, Tuojie Yang

**Affiliations:** 1 College of Chemistry and Chemical Engineering, Henan Polytechnic University, Jiaozuo 454003, China; 2 College of Food Science and Engineering, Central South University of Forestry and Technology, Changsha 410004, China

**Keywords:** microwave-assisted extraction, protic ionic liquids (PILs), polarity, *Rheum palmatum* L.

## Abstract

*Rheum palmatum* L. (*R. palmatum* L.) is a traditional Chinese herb and food, in which rhein and emodin are the main bioactive components. The extraction of the two compounds from *R. palmatum* L. is, thus, of great importance. In this work, protic ionic liquids (PILs) were applied in the microwave-assisted extraction (MAE) of rhein and emodin from *R. palmatum* L., which avoids the toxicity of organic solvents. The results of the present study indicate that PILs possessing higher polarity exhibit higher extraction ability due to their stronger absorption ability for microwave irradiation. Compared with conventional solvents, such as methanol, trichloromethane, and deep eutectic solvents (DESs), the PIL, 1-butyl-3-himidazolium methanesulfonate ([BHim]MeSO_3_) reported herein is more efficient. The selected extraction conditions of liquid–solid ratio, microwave irradiation time, microwave irradiation power, and PIL concentration were 40 g·g^−1^, 50 s, 280 W, and 80%, respectively. Under the selected conditions, the extraction yields of rhein and emodin were 7.8 and 4.0 mg·g^−1^, respectively. These results suggest that PILs are efficient extraction solvents for the separation of active components from natural products.

## 1. Introduction

*Rheum palmatum* L., a well-known traditional Chinese herb and food, is used to treat various diseases and adverse conditions, such as high fever, intense sweating, constipation, and abdominal pain [1,2]. Rhein and emodin, as the major components of *R. palmatum* L., were investigated in depth and are widely used in pharmaceuticals, functional foods, and natural yellow dyes because of their excellent bioactivities [3,4]. Consequently, an efficient green extraction method should be developed to extract rhein and emodin from *R. palmatum* L. The traditional extraction of rhein and emodin from *R. palmatum* L. is usually carried out using many time-consuming and inefficient conventional extraction techniques, such as marinated extraction (ME), heat reflux extraction (HRE), and Soxhlet extraction [5]. Modern extraction techniques such as microwave-assisted extraction (MAE) provide an efficient alternative with the advantages of higher extraction efficiency, faster extraction processes, lower solvent consumption etc. [6]. However, the remarkable deficiency of MAE is that the abundant volatile organic solvents used in the extraction process have detrimental effects on the environment and human health. Ionic liquids (ILs), constituting organic cations and inorganic anions with unique properties, such as a low melting temperature, high thermal stability, wide liquid phase range, and low vapor pressure, were used in the extraction of natural products [7,8,9]; however, 1,3-dialkylimidazolium-based ILs used in previous extraction processes entail a high cost [10,11,12]. Therefore, the use of green and economic solvents instead of hazardous solvents is one of the most important considerations during extraction [13,14]. In this context, Procopio and Nardi’s group used the MAE method with an eco-sustainable solvent, water, as the extraction solvent to extract oleuropein from olive leaves [15,16]. Another interesting work from the same research group also reported the extraction of demethyloleuropein from ripened drupes of the Leccino cultivar using methanol maceration at room temperature [17].

Additionally, Wu et al. proposed an ultrasound-assisted extraction (UAE) method for the extraction of total anthraquinones from *R. palmatum* L. using natural deep eutectic solvents (DESs) [18]. The reported DES, consisting of lactic acid, glucose, and water (LGH), was highly efficient in extracting anthraquinones from *R. palmatum* L., and it possesses many noteworthy advantages, such as low cost, easy preparation, biodegradability, pharmaceutically acceptable toxicity, and sustainability [19,20]. Inspired by their work, we tried to develop a fast, highly effective, environmentally benign, and low-cost method for the extraction of *R. palmatum* L. through changing DESs into protic ionic liquids (PILs) and using the MAE method instead of UAE. The MAE technique was adopted due to its unique properties, such as high efficiency, simple operation, shorter extraction time, and low cost [21,22]. The PILs were selected because of their advantageous property distinguishing them from other ILs. Firstly, the PILs have a strong ability to absorb microwave irradiation due to their highly polar nature [23,24]. Secondly, the synthesis of PILs is simple and easy, via equimolar mixing of acids and bases, possessing the advantages of low cost and easy preparation.

The aim of this work was, thus, to develop a fast and effective PIL MAE method for the extraction of rhein and emodin from *R. palmatum* L. As far as we know, PIL MAE methods used in the extraction of target products from *R. palmatum* L. are seldom reported. The effects of different extraction parameters, namely, the structure of the PILs, microwave irradiation power, PIL concentration, extraction time, and liquid–solid ratio, on the extraction properties were studied. The investigated technique showed the excellent extraction of rhein and emodin at a low cost.

## 2. Results and Discussion

### 2.1. The Choice of PIL

**Figure 1:**
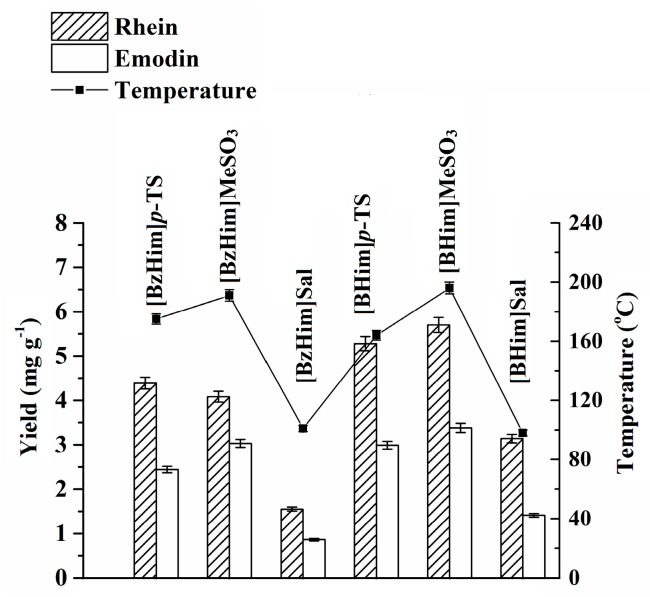
Extraction ability of different protic ionic liquids (PILs). Liquid–solid ratio, 20 g·g^−1^; microwave irradiation power, 280 W; microwave irradiation time, 70 s; *C*_IL_ = 80%.

In this work, six PILs with different chemical structures were selected, and their extraction ability for rhein and emodin was studied. The results shown in Figure 1 indicate that the PILs with salicylate as the anion exhibited a lower extraction ability, e.g., *E*([BzHim]MeSO_3_) > *E*([BzHim]Sal). It is known that ILs with a high polarity have a stronger microwave absorption ability [25,26], leading to a higher extraction temperature and higher extraction efficiency. Therefore, the lower extraction ability of salicylate-based PILs may be attributed to their lower polarity. As reported in the literature [27,28,29], the ^1^H-NMR chemical shifts (*δ*) of the hydrogen atom in the 2-position (H2) of imidazolium are related to the polarity of ILs, i.e., ILs with a stronger polarity have a higher chemical shift. As shown in Section 3.2, the *δ* values of H2 of ILs with MeSO_3_^−^ and *p*-TS^−^ (*p*-toluenesulfonate) as anions were in the range of 9.136 to 9.296, and those of ILs with salicylate as the anion were within 8.541–8.629. This suggests that salicylate-based ILs have a lower polarity and, thus, exhibit poor extraction ability, which is in good agreement with the above observation (Figure 1). Furthermore, the extraction ability of [BHim]MeSO_3_ was slightly higher than that of [BHim]*p*-TS, which may be ascribed to the fact that [BHim]MeSO_3_ has a lower molecular weight and, thus, a higher molar concentration. Thus, [BHim]MeSO_3_ was selected as the extraction solvent and used in the subsequent experiments.

### 2.2. Selection of Extraction Conditions

**Figure 2:**
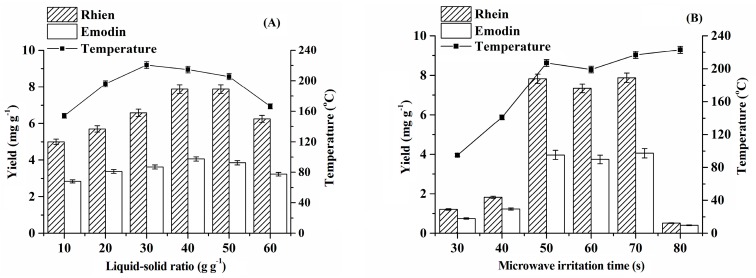
Influence of liquid–solid ratio (**A**) (microwave irradiation power, 280 W; microwave irradiation time, 70 s; *C*_IL_ = 80%) and microwave irradiation time (**B**) (microwave irradiation power, 280 W; liquid–solid ratio, 40 g·g^−1^; *C*_IL_ = 80%) on the extraction efficiency.

**Figure 3:**
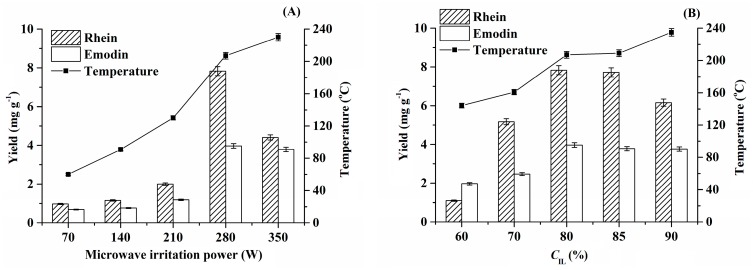
Influence of microwave irradiation power (**A**) (microwave irradiation time, 50 s; liquid–solid ratio, 40 g·g^−1^; *C*_IL_ = 80%) and ionic liquid (IL) concentration (**B**) (microwave irradiation power, 280 W; liquid–solid ratio, 40 g·g^−1^; microwave irradiation time, 50 s) on the extraction efficiency.

In this work, the influence of the extraction conditions, such as liquid–solid ratio, extraction time, microwave irradiation power, and IL concentration on the extraction efficiency was studied systematically, and the results are shown in Figure 2 and Figure 3. As shown in Figure 2A, the extraction efficiencies of rhein and emodin rose upon increasing the liquid–solid ratio from 10 to 40 g·g^−1^, remained constant within 40–50 g·g^−1^, and slightly decreased at 60 g·g^−1^. Thus, a liquid–solid ratio of 40 g·g^−1^ was selected and used in the following studies.

The results shown in Figure 2B suggest that, within 30–50 s, a longer microwave irradiation time resulted in a higher extraction efficiency, and the extraction efficiency remained constant within 50–70 s. However, when the microwave irradiation time reached 80 s, the extraction efficiencies of rhein and emodin were rather poor, which can be attributed to the following fact: a longer microwave irradiation time (80 s) means that the target compounds must endure longer heating times, which may cause the partial decomposition of rhein and emodin. To confirm this deduction, the recoveries of rhein and emodin were determined (spiked level, 8.0 mg·g^−1^ for rhein and 4.0 mg·g^−1^ for emodin) under 80 s of microwave irradiation time. The experimental results indicated that the spiked recoveries of rhein and emodin were only 8.0% and 16.8%, respectively, which means that most of the two target compounds were decomposed. Based on the above discussion, 50 s of microwave irradiation time was chosen for the subsequent experiments.

The effect of microwave irradiation power on the extraction efficiency is illustrated in Figure 3A. As can be seen, the extraction efficiencies of rhein and emodin increased upon increasing the microwave irradiation power from 70 W to 280 W, which can be ascribed to the fact that high-power microwave irradiation leads to a higher extraction temperature and, thus, a higher extraction efficiency. When 350 W was adopted, the extraction efficiency decreased remarkably, which could also be attributed to the partial decomposition of rhein and emodin due to excessive temperature. Based on these results, 280 W was adopted as the microwave irradiation power for the following studies.

The experimental results illustrated in Figure 3B show that a higher IL concentration (60–85%) led to a higher extraction efficiency, and 90% IL resulted in a significant decrease in the extraction efficiency, which was ascribed to the excessive extraction temperature at 90% IL. In view of the fact that 80% IL exhibited a higher extraction ability, it was, thus, selected for the subsequent experiments.

### 2.3. Comparison with Reported Methods

Recently, various extraction methods, such as UAE/LGH (where LGH is a solution of lactic acid, glucose, and water) [18], UAE/84% methanol [30] and HRE/methanol–trichloromethane [31] were used to extract rhein and emodin. Therefore, comparative experiments were conducted to investigate their ability for the extraction of rhein and emodin from the *R. palmatum* L. powder used in this work. Furthermore, the contrasting experiment of MAE/LGH was also done in this work. The results shown in Table 1 indicate that, compared with UAE and HRE, the MAE method is faster and more effective. It is worth mentioning that, under the selected experimental conditions, the extraction ability of MAE/[BHim]MeSO_3_ was the best in terms of the contents of rhein (7.8 mg·g^−1^) and emodin (4.0 mg·g^−1^), better than the highest data obtained using HRE method (7.3 mg·g^−1^ and 3.5 mg·g^−1^, respectively) [31]. Additionally, the product contents (rhein 2.2 mg·g^−1^ and emodin 0.87 mg·g^−1^) of MAE/LGH were remarkably lower than those of MAE/[BHim]MeSO_3,_ which can be ascribed to the fact that [BHim]MeSO_3_ is composed of ions and is more polar than LGH. Thus, the extraction temperature of MAE/[BHim]MeSO_3_ was higher than MAE/LGH, resulting in the higher extraction ability of MAE/[BHim]MeSO_3_. Above all, the extraction ability of PIL/MAE proposed by this work was faster and more effective than the previously reported extraction modes.

### 2.4. Recyclability of [BHim]MeSO_3_ and the Purification of Rhein and Emodin

To reuse [BHim]MeSO_3_, ethyl acetate is used to extract rhein and emodin from the [BHim]MeSO_3_ solution after extraction. After filtration to remove *R. palmatum* L., the recovered [BHim]MeSO_3_ can be used for the next extraction cycle without losing its extraction ability. Rhein and emodin in the ethyl acetate phase were purified using column chromatography using silica gel as the stationary phase and a mixture of ethyl acetate and petroleum ether as the eluent (30:70, *v*/*v*). The purity (determined by the HPLC method as described in Section 3) of the resultant rhein and emodin was 92.5% and 93.2%, respectively.

## 3. Materials and Methods

### 3.1. Reagents

Emodin (90%), rhein (98%), salicylic acid (99.5%), methanesulfonic acid (99%), and l-lactic acid (90%) were purchased from Aladdin Bio-Chem Technology Co., Ltd. (Shanghai, China). Furthermore, *p*-toluenesulfonic acid monohydrate (*p*-TSA·H_2_O, 98%) was supplied by Tokyo Chemical Industry Co., Ltd. (Tokyo, Japan), while 1-benzylimidazole (98%) and 1-butylimidazole (98%) were obtained from Zhongkai Chem. Co., Ltd. (Changzhou, China). Ultrapure water (resistivity, 18.2 MΩ∙cm) was used in the experiments (Aquapro purification system, Aquapro International Co., Ltd., Dover, DE, USA). All the other reagents used were of analytical grade unless stated otherwise. *R. palmatum* L. powder (100 mesh) was obtained from Xuanqing Biotechnology Co., Ltd. (Kunming, China).

### 3.2. Synthesis of PILs

To prepare 1-benzyl-3*H*-imidazolium salicylate ([BzHim]Sal), 0.1 mol of 1-benzylimidazole, 0.1 mol of salicylic acid, and 30 mL of methanol were mixed while stirring until a homogeneous solution was formed. After removing the solvent under vacuum, a light-yellow liquid was obtained. The PIL was identified by ^1^H-NMR (400 MHz, DMSO-*d*_6_ (hexadeuterodimethyl sulfoxide)) with the following chemical shifts (*δ*): 5.328 (s, 2H), 6.790–6.851 (m, 2H), 7.320–7.401 (m, 7H), 7.484 (s, 1H), 7.790–7.813 (m, 1H), 8.541 (s, 1H), and 12.725 (s, 1H).

The preparation of 1-benzyl-3*H*-imidazolium methanesulfonate ([BzHim]MeSO_3_) followed the same procedure as for [BzHim]Sal described above, except methanesulfinic acid was used instead of salicylic acid. The product ([BzHim]MeSO_3_) was a white solid with a melting point of 36–38 °C. The chemical shifts (*δ*) of ^1^H-NMR (400 MHz, DMSO-*d*_6_) were as follows: 2.349 (s, 3H), 5.451 (s, 2H), 7.380–7.430 (m, 5H), 7.707 (s, 1H), 7.806 (s, 1H), and 9.296 (s, 1H).

The preparation of 1-benzyl-3*H*-imidazolium *p*-toluenesulfonate ([BzHim]*p*-TS) followed the same procedure as for [BzHim]Sal described above, except *p*-toluenesulfonic acid was used instead of salicylic acid. The PIL, [BzHim]*p*-TS, was a white solid with a melting point of 106–108 °C. The chemical shifts (*δ*) of ^1^H-NMR (400 MHz, DMSO-*d*_6_) were as follows: 2.287 (s, 3H), 5.438 (s, 2H), 7.106–7.125 (d, 2H), 7.395–7.422 (m, 5H), 7.479–7.499 (d, 2H), 7.697–7.705 (t, 1H), 7.794–7.801 (t, 1H), and 9.273 (s, 1H).

The synthesis of 1-butyl-3*H*-imidazolium-based PILs ([BHim]-based PILs) was similar to that of [BzHim]-based ones (equimolar mixing of 1-butylimidazole and a specific acid (*p*-toluenesulfonic acid, methanesulfinic acid, and salicylic acid)).

The PIL, [BHim]Sal, was a colorless liquid. The chemical shifts (*δ*) of ^1^H-NMR (400 MHz, DMSO-*d*_6_) were as follows: 0.852–0.888 (t, 3H), 1.171–1.245 (m, 2H), 1.692–1.765 (m, 2H), 4.082–4.118 (t, 3H), 6.756–6.815 (m, 2H), 7.299–7.342 (m, 1H), 7.420 (s, 1H), 7.553 (s, 1H), 7.787–7.810 (m, 1H), 8.629 (s, 1H), and 13.190 (s, 1H).

The PIL, [BHim]*p*-TS, was a white solid with a melting point of 58–59 °C. The chemical shifts (*δ*) of ^1^H-NMR (400 MHz, DMSO-*d*_6_) were as follows: 0.874–0.910 (t, 3H), 1.208–1.264 (m, 2H), 1.735–1.791 (m, 2H), 2.288 (s, 3H), 4.163–4.199 (t, 2H), 7.108–7.129 (d, 2H), 7.478–7.498 (d, 2H), 7.690 (s, 1H), 7.795 (s, 1H), and 9.136 (s, 1H).

The PIL, [BHim]MeSO_3_, was a white solid with a melting point of 68–70 °C. The chemical shifts (*δ*) of ^1^H-NMR (400 MHz, DMSO-*d*_6_) were as follows: 0.880–0.916 (t, 3H), 1.218–1.274 (m, 2H), 1.746–1.802 (m, 2H), 2.339 (s, 3H), 4.175–4.212 (t, 2H), 7.698 (s, 1H), 7.804 (s, 1H), and 9.151 (s, 1H).

**Figure 4:**
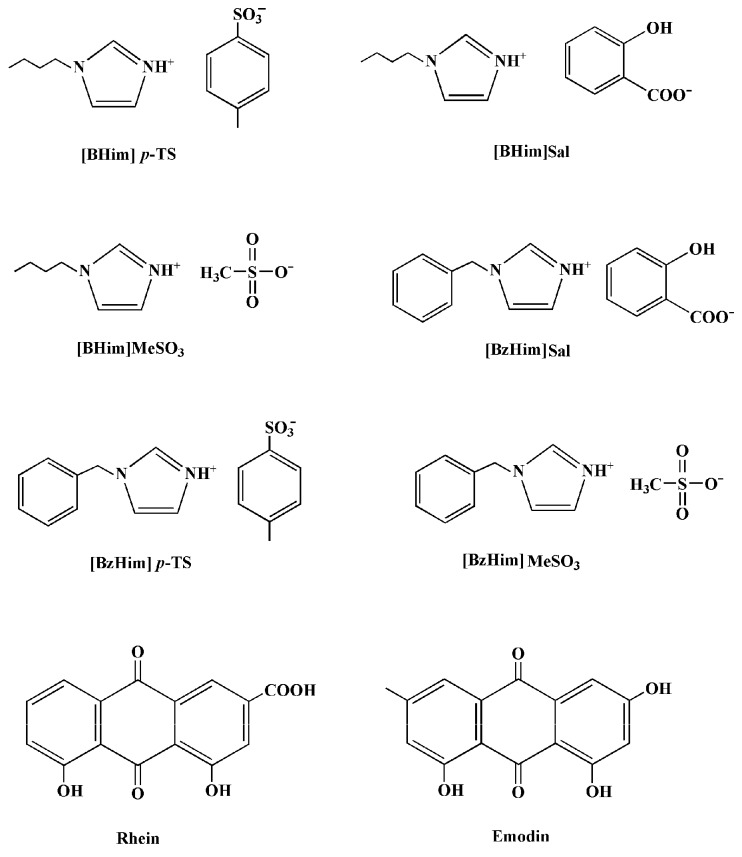
Chemical structures of PILs, rhein, and emodin.

The chemical structures of the PILs, rhein, and emodin are shown in Figure 4.

### 3.3. Microwave-Assisted Extraction (MAE)

Briefly, a mixture of 0.1 g of *R. palmatum* L. powder and 2.0 g of PIL (80%, wt.%) was heated in a microwave oven (model G70F20CPIII-TK(W0), Guangdong Galanz Microwave Oven and Electrical Appliances Manufacturing Co., Ltd., Foshan, China). The microwave irradiation power and time were in the range of 70 W to 350 W and 30 s to 80 s, respectively. Extraction temperature (60 °C to 235 °C) was measured by a non-contact infrared thermometer (model, UT300A, Uni-Trend technology Co., Ltd., Dongguan, China). After extraction, the resultant mixture was diluted to 10 mL with ethanol and filtrated by a nylon membrane (pore size, 0.45 μm). The contents of emodin and rhein in ethanol solution were analyzed by an Agilent 1200 high-performance liquid chromatograph (HPLC, Agilent Technologies, Santa Clara, CA, USA) equipped with a degasser, an autosampler, and a variable wavelength detector. The HPLC conditions were as follows: column temperature, 30 °C; injection volume, 5.0 μL; detection wavelength, 254 nm; separation column, ZORBAX Eclipse XDB-C18 column (4.6 mm × 150 mm, 5 μm, Agilent Technologies, Santa Clara, USA); flow rate, 1.0 mL∙min^−1^; mobile phase, a mixture of acetonitrile and 0.1% (*v*/*v*) acetic acid aqueous solution (40% (*v*/*v*) acetonitrile); run time, 15 min.

### 3.4. Heat Reflux Extraction (HRE)

This extraction procedure was conducted according to the standard method of the Chinese Pharmacopoeia [31]. Typically, 0.15 g of *R. palmatum* L. powder was mixed with 25 mL of methanol, and the resultant mixture was heated to reflux for 1.0 h. After cooling and filtration, 5.0 mL of the filtrate was withdrawn and evaporated under vacuum to remove the solvent; then, 10 mL of 8% HCl solution was added and subsequently sonicated for 2.0 min. After that, 10 mL of trichloromethane was added and heated to reflux for 1.0 h. After cooling, the trichloromethane phase was withdrawn, and the aqueous phase was washed three times, each time with 10 mL of trichloromethane. The trichloromethane phase was combined and evaporated under vacuum to remove the solvent. The resultant residue was dissolved with methanol and filtrated by a nylon membrane (pore size, 0.45 μm) before HPLC analysis.

### 3.5. Ultrasound-Assisted Extraction (UAE)

The UAE procedure was the same as that reported in the literature [30], with the following parameters: extraction solvent, 84% (*v*/*v*) methanol aqueous solution; liquid–solid ratio, 15:1 (mL/g); extraction time, 33 min; extraction temperature, 67 °C; extraction power, 250 W. After extraction, the filtrate was collected, and the residue was extracted again (two times) with the same volume of fresh solvent. The UAE method with LGH as the extraction solvent was conducted in a similar way under the conditions recommended by the reported work [18]. The contents of rhein and emodin were determined by the aforementioned HPLC method.

## 4. Conclusions

The present work developed a fast and effective MAE technique to extract rhein and emodin from *R. palmatum* L. using PILs as extraction solvents. The key advantage of PILs is that they are easy to prepare at low cost. Experimental results suggest that the PILs owning a higher polarity have a stronger microwave absorption ability and, thus, exhibit a higher extraction ability. The extraction ability of the PIL, [BHim]MeSO_3_, was higher than that of DES (LGH), and the extraction ability of the proposed PIL-based MAE was superior to that of UAE and HRE. After extraction, the PIL, [BHim]MeSO_3_, could be recycled via back extraction without losing its extraction ability. All the above results drive to the conclusion that PIL-based MAE is an efficient, rapid, simple, and economic method to extract rhein and emodin from *R. palmatum* L.

## Figures and Tables

**Table 1:** Comparison between microwave-assisted extraction (MAE) with [BHim]MeSO_3_ and other extraction methods. UAE—ultrasound-assisted extraction.

Extraction Method	Extraction Time	Content (mg∙g^−1^)	
		Rhein	Emodin
UAE/LGH ^a^	1.5 h	1.6	1.4
UAE/84% methanol ^b^	33 min	1.9	1.1
HRE/methanol–trichloromethane ^c^	2 h	7.3	3.5
MAE/LGH ^d^	50 s	2.2	0.87
MAE/80% [BHim]MeSO_3_^e^	50 s	7.8	4.0

^a^ LGH, lactic acid–glucose–water = 5:1:3. ^a,b,c^ Experiments were conducted under the conditions recommended by References [15,27,28], respectively. ^d,e^ Experiments were carried out under the conditions discussed above.

## References

[1] Liu Y., Li L., Xiao Y.Q., Yao J.Q., Li P.Y., Yu D.R., Ma Y.L. (2016). Global metabolite profiling and diagnostic ion filtering strategy by LC-QTOF MS for rapid identification of raw and processed pieces of *Rheum palmatum* L.. Food Chem..

[2] Aichner D., Ganzera M. (2015). Analysis of anthraquinones in rhubarb (*Rheum palmatum* and *Rheum officinale*) by supercritical fluid chromatography. Talanta.

[3] Zhou Y., Zhang J., Tang R.C., Zhang J. (2015). Simultaneous dyeing and functionalization of silk with three natural yellow dyes. Ind. Crops. Prod..

[4] Khan S.A., Shahid-ul-Islam, Shahid M., Khan M.I., Yusuf M., Rather L.J., Khan M.A., Mohammad F. (2015). Mixed metal mordant dyeing of wool using root extract of *Rheum emodi* (Indian Rhubarb/Dolu). J. Nat. Fibers.

[5] Huang W., Xue A., Niu H., Jia Z., Wang J.W. (2009). Optimised ultrasonic-assisted extraction of flavonoids from Folium eucommiae and evaluation of antioxidant activity in multi-test systems in vitro. Food Chem..

[6] Sun C., Liu H.Z. (2008). Application of non-ionic surfactant in the microwave-assisted extraction of alkaloids from *Rhizoma coptidis*. Anal. Chim. Acta.

[7] Han Y., Yang C., Zhou Y., Han D., Yan H. (2017). Ionic liquid–hybrid molecularly imprinted material–filter solid-Phase extraction coupled with HPLC for determination of 6-benzyladenine and 4-chlorophenoxyacetic acid in bean sprouts. J. Agric. Food Chem..

[8] Liang R., Bao Z., Su B., Xing H., Yang Q., Yang Y., Ren Q. (2013). Feasibility of ionic liquids as extractants for selective separation of vitamin D3 and tachysterol3 by solvent extraction. J. Agric. Food Chem..

[9] Cao Y., Xing H., Yang Q., Bao Z., Su B., Yang Y., Ren Q. (2012). Separation of soybean isoflavone aglycone homologues by ionic liquid-based extraction. J. Agric. Food Chem..

[10] Khezeli T., Daneshfar A., Sahraei R. (2016). A green ultrasonic-assisted liquid-liquid microextraction based on deep eutectic solvent for the HPLC-UV determination of ferulic, caffeic and cinnamic acid from olive, almond, sesame and cinnamon oil. Talanta.

[11] Amiri-Rigi A., Abbasi S. (2016). Microemulsion-based lycopene extraction: Effect of surfactants, co-surfactants and pretreatments. Food Chem..

[12] Pawłowska B., Feder-Kubis J., Telesiński A., Biczak R. (2019). Biochemical responses of wheat seedlings on the introduction of selected chiral ionic liquids to the soils. J. Agric. Food Chem..

[13] Tobiszewski M., Mechlinska A., Namiesnik J. (2010). Green analytical chemistry—Theory and practice. Chem. Soc. Rev..

[14] Prat D., Pardigon O., Flemming H.W., Letestu S., Ducandas V., Isnard P., Guntrum E., Senac T., Ruisseau S., Cruciani P. (2013). Sanofi’s solvent selection guide: A step toward more sustainable processes. Org. Process Res. Dev..

[15] Procopio A., Alcaro S., Nardi M., Oliverio M., Ortuso F., Sacchetta P., Sindona G. (2009). Synthesis, biological evaluation, and molecular modeling of oleuropein and its semisynthetic derivatives as cyclooxygenase inhibitors. J. Agric. Food Chem..

[16] Nardi M., Bonacci S., Cariati L., Costanzo P., Oliverio M., Sindona G., Procopio A. (2017). Synthesis and antioxidant evaluation of lipophilic oleuropein aglycone derivatives. Food Funct..

[17] Nardi M., Bonacci S., De Luca G., Maiuolo J., Oliverio M., Sindona G., Procopio A. (2014). Biomimetic synthesis and antioxidant evaluation of 3,4-DHPEA-EDA [2-(3,4-hydroxyphenyl) ethyl (3S, 4E)-4-formyl-3-(2-oxoethyl)hex-4-enoate]. Food Chem..

[18] Wu Y.C., Wu P., Li Y.B., Liu T.C., Zhang L., Zhou Y.H. (2018). Natural deep eutectic solvents as new green solvents to extract anthraquinones from *Rheum palmatum* L.. RSC Adv..

[19] Dai Y., van Spronsen J., Witkamp G.J., Verpoorte R., Choi Y.H. (2013). Natural deep eutectic solvents as new potential media for green technology. Anal. Chim. Acta.

[20] Wei Z.F., Wang X.Q., Peng X., Wang W., Zhao C.J., Zu Y.G., Fu Y.J. (2015). Fast and green extraction and separation of main bioactive flavonoids from *Radix Scutellariae*. Ind. Crops Prod..

[21] Wang S.Y., Yang L., Zu Y.G., Zhao C.J., Sun X.W., Zhang L., Zhang Z.H. (2011). Design and performance evaluation of ionic-liquids-based microwave-assisted environmentally friendly extraction technique for camptothecin and 10-hydroxycamptothecin from Samara of *Camptotheca acuminata*. Ind. Eng. Chem. Res..

[22] Chi Y., Zhang Z., Li C., Liu Q., Yan P., Welz-Biermann U. (2011). Microwave-assisted extraction of lactones from *Ligusticum chuanxiong* Hort. using protic ionic liquids. Green Chem..

[23] Greaves T.L., Drummond C.J. (2008). Protic ionic liquids: Properties and applications. Chem. Rev..

[24] Hu H., Yang H., Huang P., Cui D., Peng Y., Zhang J., Lu F., Lian J., Shi D. (2010). Unique role of ionic liquid in microwave-assisted synthesis of monodisperse magnetite nanoparticles. Chem. Commun..

[25] Arsalani N., Zare P., Namazi H. (2009). Solvent free microwave assisted preparation of new telechelic polymers based on poly(ethylene glycol). Express Polym. Lett..

[26] Yang F., Gong J., Yang E., Guan Y., He X., Liu S., Zhang X., Deng Y. (2019). Microwave-absorbing properties of room-temperature ionic liquids. J. Phys. D Appl. Phys..

[27] Spange S., Lungwitz R., Schade A. (2014). Correlation of molecular structure and polarity of ionic liquids. J. Mol. Liq..

[28] Chen S., Izgorodina E.I. (2017). Prediction of ^1^H NMR chemical shifts for clusters of imidazolium-based ionic liquids. Phys. Chem. Chem. Phys..

[29] Lin S.T., Ding M.F., Chang C.W., Lue S.S. (2004). Nuclear magnetic resonance spectroscopic study on ionic liquids of 1-alkyl-3-methylimidazolium salts. Tetrahedron.

[30] Zhao L.C., Liang J., Li W., Cheng K.M., Xia X., Deng X., Yang G.L. (2011). The use of response surface methodology to optimize the ultrasound-assisted extraction of five anthraquinones from *Rheum palmatum* L.. Molecules.

[31] Chinese Pharmacopoeia Commission (2015). Pharmacopoeia of the People’s Republic of China.

